# Effects of *Pasteurella multocida* on Histopathology, miRNA and mRNA Expression Dynamics in Lung of Goats

**DOI:** 10.3390/ani12121529

**Published:** 2022-06-13

**Authors:** Wencan Zhang, Zizhuo Jiao, Huixian Huang, Yanru Wu, Haotian Wu, Zhiyong Liu, Zhenxing Zhang, Qi An, Yiwen Cheng, Si Chen, Churiga Man, Li Du, Fengyang Wang, Qiaoling Chen

**Affiliations:** Hainan Key Laboratory of Tropical Animal Reproduction & Breeding and Epidemic Disease Research, Engineering Key Lab of Haikou, College of Animal Science and Technology, Hainan University, Haikou 570228, China; zhangwencan666@outlook.com (W.Z.); jiaozizhuo1998@outlook.com (Z.J.); huixian9626@163.com (H.H.); wwsz1227@163.com (Y.W.); 1203502489a@gmail.com (H.W.); johnny_lzy@163.com (Z.L.); zxzhang23@163.com (Z.Z.); i.angie@outlook.com (Q.A.); cyw0506@outlook.com (Y.C.); chensi.ruth@hotmail.com (S.C.); manchuriga@163.com (C.M.); kych2008dl@163.com (L.D.); fywang68@163.com (F.W.)

**Keywords:** lung, histopathological changes, *Pasteurella multocida*, miRNA, mRNA

## Abstract

**Simple Summary:**

*Pasteurella multocida* (Pm) infection can cause pasteurellosis in goats. Currently, the mechanisms of Pm infection in goats are limited. In this study, the effects of Pm infection to goats were characterized from the aspects of histopathology, miRNA and mRNA expression dynamics in lung of goats infected for 1, 2, 5 and 7 days and the clinical symptoms of animals. We further revealed the potential role of differentially expressed RNAs, which are commonly involved in and regulate the host immune reaction to the Pm infection. The differentially expressed genes were mapped to marker genes of the immune cells in the lung, which further confirmed the cells detected by the histopathological method.

**Abstract:**

*Pasteurella multocida* (Pm) infection causes severe respiratory disease in goats. We investigated the effects of the Pm infection intratracheally on the histopathology, miRNA and mRNA expression dynamics in the lung of goats infected for 1, 2, 5 and 7 days. Pm infection caused fever, which significantly (*p* < 0.05) increased the body temperature of the goats from day 1 to 5. Haemotoxylin–eosin staining of the infected lung tissue showed characteristics of suppurative pneumonia with inflammatory cells infiltration and the lung structure destruction. During the Pm infection of the goats, compared with the control group, there were 3080, 3508, 2716 and 2675 differentially expressed genes and 42, 69, 91 and 108 significantly expressed miRNAs (|log_2_Fold Change| > 1, *p* < 0.05) in the Pm_d1, Pm_d2, Pm_d5 and Pm_d7 groups, respectively. Five miRNAs and nine immune-related genes were selected for confirmation by reverse transcription–polymerase chain reaction. The results indicated that the expression patterns of the miRNAs and genes were consistent with those determined by next-generation sequencing. The differentially expressed genes were enriched in cytokine–cytokine receptor interaction, cell adhesion molecules, complement and coagulation cascades, tight junction and phagosome Kyoto Encyclopedia of Genes and Genomes pathways and cytokine production, leukocyte migration, myeloid leukocyte migration, cell periphery, plasma membrane, extracellular region part, extracellular region and other Gene Ontology terms. The differentially expressed genes were mapped to marker genes in human and mouse lung cells. The results showed the presence of some marker genes of the immune cells. Compared with the CK group, five miRNAs and 892 common genes were differentially expressed in the Pm_d1, Pm_d2, Pm_d5 and Pm_d7 groups. The target relationships between the common 5 miRNAs and 892 differentially expressed genes were explored and the miRNAs involved in the host immune reaction may act through the target genes. Our study characterized goats’ reaction in the lung from histopathological and molecular changes upon Pm infection, which will provide valuable information for understanding the responses in goats during Pm infection.

## 1. Introduction

*Pasteurella multocida* (Pm) is a highly versatile opportunistic pathogen that causes infections in a wide range of domestic and wild animals and humans [1]. It is found mainly in the oral cavity, nasopharyngeal and upper respiratory tract of animals [2]. Goats are distributed worldwide and provide meat and milk for human consumption and fiber [3]. Pasteurellosis causes two significant diseases in goats: pneumonic pasteurellosis and systemic pasteurellosis [4]. The high incidence and rapid transmission of the diseases cause substantial economic losses to the goat industry.

The symptoms of Pm infection in goats usually begin with fever, lethargy, anorexia and edema with copious salivation, lacrimation, nasal discharge, coughing and dullness. These symptoms are rapidly followed by respiratory distress, septic shock with widespread hemorrhaging and death [4,5]. In addition, hematological, biochemical and pathological changes were observed [4,6]. Pm-infected goats showed a significant decrease in hemoglobin count and significant increase in white blood cells, neutrophil and monocyte counts, respectively. Infected goats revealed significantly lower concentrations of Ca and Mg but a considerably higher concentration of P in serum than healthy goats [4]. Microscopical examination of the lungs of Pm-infected goats indicated that goats with acute pneumonia showed edema and dense infiltration of neutrophils in the alveolar spaces as well as severely congested inter-alveolar capillaries [5]. Neutrophils, alveolar macrophages, fibrin and protein-rich oedematous fluid are present in the alveolar spaces. In more advanced cases, macrophages and fibrin were observed among the neutrophils [5]. Despite extensive research on the clinical, pathological, hematological and biochemical parameters of Pm-infected goats, very little is known about the molecular mechanisms of the Pm infection of goats.

High-throughput sequencing (HTS) is widely used as a screening method for differentially expressed genes (DEGs), mainly in transcriptomics, genomics, epigenomics and immunomics. Research on the application of HTS in Pm-infected goats is limited. In contrast, the disease has been widely studied in mice, pigs, poultry and other animals. Wu et al. established a mouse model for *Pasteurella pneumonia* and detected 4236 DEGs by transcriptome sequencing analysis [7]. Newborn pig tracheal epithelial (NPTr) cells were used as an in vitro model and the results found that Pm infection disrupted the barrier functions of the NPTr cells. RNA sequencing showed 30 DEGs between the Pm-infected pig tracheal epithelial cells and the control group [8]. The transcriptomes data from chicken lungs infected with two different lethality isolates Pm were used for the cell-type enrichment analysis, the results showed that immune cells were enriched in both infection groups [9]. A number of studies evaluated the effects of Pm on transcripts and host immune reaction in Pm-infected animals, but none have used HTS to analyse the miRNA and mRNA expression and function mechanism in goat.

The objective of this study, therefore, was to characterize the histopathology, miRNA and mRNA expression profiles in goat lungs from uninfected (the control group) and Pm-infected groups (the Pm group) at 1, 2, 5 and 7 days. In addition, we also evaluated the potential relationship between the miRNAs and mRNA expression and explored their possible functions in the Pm-caused inflammation and host immune response.

## 2. Materials and Methods

### 2.1. Bacterial Culture

*Pasteurella multocida* strain HN01, previously isolated from the lungs of Hainan Black goats, was stored in our laboratory (GenBank accession No. cp037861). Pm HN01 isolates were revived for the current study as described previously [10]. Briefly, the bacteria were confirmed by a 16s rRNA polymerase chain reaction (PCR). Colonies were picked and cultured in tryptic soy broth, supplemented with 5% (*v/v*) bovine serum, at 37 °C overnight for 12 h with shaking at 220 rpm. For the challenge assays, goats were intratracheally infected with 1 × 10^9^ CFU of Pm HN01 in 2 mL of sterilized phosphate-buffered saline (PBS, pH 7.2).

### 2.2. Animal Management and Sampling

**Figure 1:**
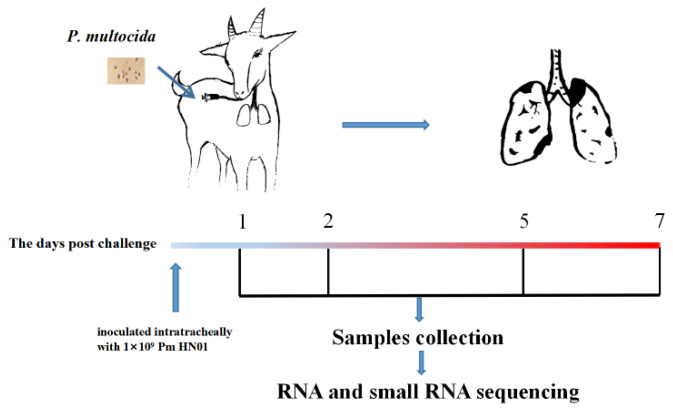
The experimental design. A total of 20 female goats were divided into five treatment groups (4 in each group): (1) an uninfected negative control group (CK: control group); (2) a group intratracheally infected with Pm HN01 for 1 day (Pm_d1 group); (3) an infected group challenged with Pm HN01 for 2 days (Pm_d2 group); (4) an infected group challenged with Pm HN01 for 5 days (Pm_d5 group); (5) an infected group challenged with Pm HN01 for 7 days (Pm_d7 group).

A total of 20 clinically healthy 3-month-old female Hainan Black goats weighing 18 ± 2 kg (mean ± SE) were purchased from a local goat farm (HuaRun Farm, Wanning City, Hainan Province, China) and used for this study. Nasal swabs and blood were collected every 3 days for 1 month before purchase to ensure that the goats were free of Pm and *Brucella* infection. The goats were allocated randomly to 5 groups (*n* = 4 per group) as follows: (1) an uninfected control group (CK group) and (2) infected groups challenged with Pm for 1 d (Pm_d1 group), 2 d (Pm_d2 group), 5 d (Pm_d5 group) and 7 d (Pm_d7 group) (Figure 1). All the goats were housed in separate sterile pens with age-appropriate temperature and humidity levels during the test period. They were fed a typical standard control diet with free access to food and water.

As shown in Figure 1, goats in the infected group were infected via intra-trachea inoculation with 2 mL (1 × 10^9^ CFU) of Pm HN01 bacterial suspension. Goats in the CK group were inoculated with 2 mL of PBS. The goats were closely monitored throughout the experimental period for clinical signs of infection. Rectal temperatures were collected daily from each goat during the study period to detect signs of Pm infection. Three challenged goats died from the acute disease within 12 h post-inoculation. The other goats were euthanized 1, 2, 5 and 7 days post-Pm-challenge. Lung tissue was collected from each goat in the Pm groups and the corresponding regions of the lungs of goats from the CK group and divided into two aliquots. One aliquot was stored in liquid nitrogen at −80 °C until total RNA and DNA extraction. At the same time, the other was fixed in 4% (*v/v*) paraformaldehyde for pathological examination following hematoxylin and eosin staining. All experimental protocols were approved by the Academic Committee of the College of Animal Science and Technology of Hainan University based on the regulations on the use of the experimental animals and the institutional safety procedures.

### 2.3. Detection of Pm in the Lungs of the Animals

To detect if the Pm survived in the lung of animals, the DNA of lung tissue was extracted following the instructions of a DNA extraction kit (Tiangen, Beijing, China). PCR for the validation of the Pm HN01 strain was carried out using specific primers for the *dcbF* gene. The primers are shown in Table A1.

### 2.4. RNA and Small RNA Sequencing

Total RNA was extracted from frozen lung tissues from three goats of each group (marked as CK and Pm_d1, Pm_d2, Pm_d5 and Pm_d7) using Trizol (Invitrogen, Carlsbad, CA, USA) according to the manufacturer’s instructions. The RNA was used for miRNA and RNA sequencing on the Illumina platform in Personalbio (Personalbio, Nanjing, China). After the quality validation by Agilent 2100 (Agilent Technologies Santa Clara, CA, USA) and gel electrophoresis, the small RNA library was constructed using the TruSeq Small RNA Sample prekit (Illumina, San Diego, CA, USA). The small RNA libraries were PCR amplified for enrichment and gel purified. The quality of libraries was then tested by Agilent 2100 Bioanalyzer and quantified by Quant-iT PicoGreen dsDNA Assay kit and subjected to deep sequencing. After the quality validation and rRNA elimination, the total RNA was fragmented to 300–400 bp and reverse transcribed to synthesize cDNA with mRNA-Seq Sample Preparation Kit (Illumina, San Diego, CA, USA). The cDNA library was constructed and tested by Agilent 2100 Bioanalyzer for paired-end sequencing by next-generation sequencing on an Illumina platform (Illumina, San Diego, CA, USA).

The raw miRNAs sequencing data was filtered and used for size distribution analyses. The clean data were mapped to the goat reference genome (Capra_hircus.ARS1.dna.toplevel.fa, http://asia.ensembl.org/Capra_hircus/Info/Index (accessed on 23 December 2020)) by Bowtie program in miRDeep2 software for further annotation analyses [11]. The resulting mapped data were aligned against miRBase, piRBase and Rfam database to identify the known miRNAs, piRNAs and non-coding RNA (ncRNAs). The reads of the known miRNAs mapped to the miRBase to statistically differential expression miRNAs. The raw data from RNA-sequencing was filtered with Cutadapt (v2.7) [12] software and the remaining clean reads with high quality were mapped to the goat reference genome using HIsat2 software (http://ccb.jhu.edu/software/hisat2/index.shtml (accessed on 23 December 2020)). The mapped read count values of each gene as the original expression were counted by the HTSeq [13].

All RNA and small RNA sequencing data generated in this study are available in the NIH short read archive under accession numbers PRJNA804497 and PRJNA781464, respectively.

### 2.5. Identification of Differentially-Expressed miRNA and mRNA Molecules

The DESeq [14] package of R software was used to identify differentially expressed miRNAs and genes between the experimental and control groups and quantify expression levels of miRNAs and genes in the lung tissue of the goats after Pm infection. MiRNAs and genes with a log_2_fold change greater than 1 and a *p*-value less than 0.05 between the two groups were identified as differentially expressed. The expression data for Pm_d1, Pm_d2, Pm_d5 and Pm_d7 were normalized and compared with the CK group, respectively. The common differentially expressed miRNAs and genes were screened and used for Short Time-series Expression Miner (STEM) analysis [15].

### 2.6. Functional Annotation of DEGs

The Kyoto Encyclopedia of Genes and Genomes (KEGG) database and Gene Ontology (GO) database were used for functional annotation of the DEGs. The GO and KEGG enrichment pathway analyses were performed using R with hypergeometric test and *p*-value < 0.05 was considered significantly enriched. In addition, the DEGs list was mapped to the mouse lung cell marker gene in the cell Marker (http://biocc.hrbmu.edu.cn/CellMarker/index.jsp (accessed on 9 November 2021)) for determining the lung cell type enrichment.

### 2.7. Validation of Differentially Expressed miRNA and mRNA Molecules in the Lung Sample by qPCR

Quantitative RT-PCR was used to validate the expression of several differentially expressed miRNAs (chi-miR-21-3p, chi-miR-130b-3p, chi-miR-758, chi-miR-665 and chi-miR-335-3p) and genes (IL10, C1QTNF7, IL12RB2, TNFSF10, IL17RD, IL11, IL13RA2, IL17A and TNFAIP6). The PCR primers for the miRNAs and genes were designed with the Primer Premier 5 (PREMIER Biosoft International, Palo Alto, CA, USA) and listed in Table A1. Total RNA was converted to the first strand of cDNA and qPCR was performed using the SYBR^®^ Premix Ex Taq kit (TaKaRa Biotechnology CO., Ltd., Dalian, China) with the Bio-Rad CFX96 qPCR system (Bio-Rad, Hercules, CA, USA). The following PCR cycling conditions were used: an initial denaturation step at 95 °C for 30 s, 40 cycles at 95 °C for 10 s, annealing temperature for 30 s and 72 °C for 10 s. MiRNA expression was detected using TaqMan probe-based qPCR, as described previously [16]. The primers and TaqMan probes used for qPCR assays were designed using Beacon Designer 7 (PREMIER Biosoft International, Palo Alto, CA, USA). Sequence about the primers and probes is provided in Table A1. The reaction mixtures used for the qPCR assays were the same as those described by Chen et al. [10]. All reactions were carried out at 95 °C for 5 min, followed by 40 cycles of 95 °C for 15 s and 60 °C for 30 s. The relative expression of the target genes and miRNAs was calculated using the 2^−^^△△Ct^ method and normalized to the expression of the house-keeping genes (GAPDH and 5S rRNA). The results were expressed as fold-change relative to the average value of the control group. All the experiments were conducted independently three times.

### 2.8. Target Gene Prediction and miRNA-Genes Network Construction

To predict the interaction relationship of differentially expressed miRNAs and genes, the 3′UTR region of DEGs was used as the target sequences for searching target differentially expressed miRNAs using miranda software (http://miranda.org.uk/ (accessed on 10 January 2021)). During the analysis, only with inverse expression trend of DE miRNAs and DEGs were selected for building miRNAs-gene networks with Cytoscape 3.6.0 software [17].

### 2.9. Statistical Analysis

Data were processed with GraphPad Prism 6.0 and presented as mean ± standard error of the mean. The statistical significance of the body temperatures of the goats between the CK and Pm-infected groups was analyzed by the non-parametric Mann–Whitney U-test with R language, and *p* < 0.05 was defined as statistically significant.

## 3. Results

### 3.1. Effects of Pm Infection on the Lung of Animals

**Figure 2:**
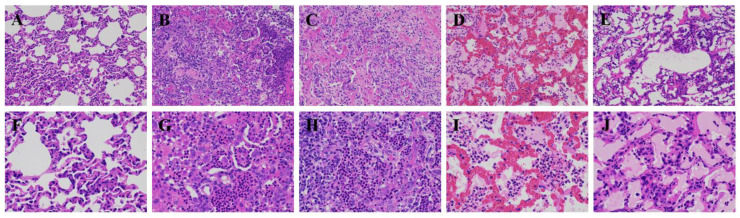
Histopathological analysis of lung lesions in the CK group (**A**,**F**) and after Pm infection 1 (**B**,**G**), 2 (**C**,**H**), 5 (**D**,**I**) and 7 (**F**,**J**) days. All the tissues were stained with hematoxylin and eosin. (**A**–**E**) Magnification, ×200; (**F**–**J**) Magnification, ×400; (**B**,**G**) The structural characteristics of the lung tissue were lost. The alveolar cavity was filled with inflammatory exudates, including neutrophils, monocytes and fibrin. (**C**,**H**) The structure of the alveolar wall was unclear; zccumulated neutrophils and macrophages were found in the alveolar cavity and some neutrophils degenerated and necrotized to form pus; lymphocyte aggregation can be seen in some areas of the alveoli. (**D**,**I**) The capillaries of the alveolar wall were highly ecchymosis and dilated. Neutrophils and edematous fluid accumulated in the alveolar cavity and red blood cells entered and presented in the alveolar cavity. (**F**,**J**) Alveolar wall thickened, alveolar epithelial cells swelled and proliferated; alveolar cavity was filled with a large amount of edema fluid, and in some of these areas there were macrophages and exfoliated and necrotic epithelial cells.

**Figure A1:**
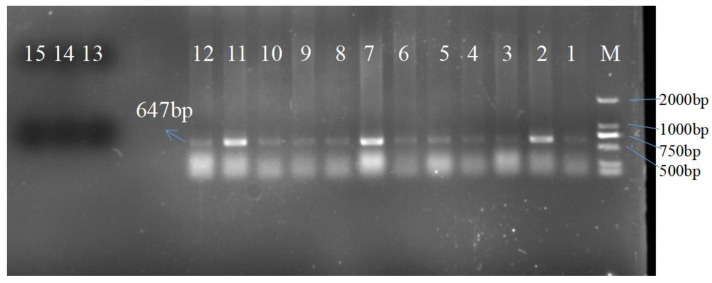
PCR-based validation of Pm in different lung samples from goats in different groups. Lane 1–12, lung samples from the Pm-infected group goats. lanes 13–15, lung samples from the CK group goats. M—Marker D2000.

Pm-infected goats exhibited fever that significantly (*p* < 0.05) increased their body temperature from day 1 to day 5, compared with the CK group (Table 1). The treatment group also showed inappetence, serous nasal discharge, salivation and breathing distress, such as coughing, sneezing and nasal discharge. Three of the challenged goats died from the acute disease within 12 h after infection. Stained lung tissue of goats in the CK and Pm-infection groups at four time points (day 1, day 2, day 5 and day 7) are shown in Figure 2. During sample collection, significant lung tissue lesions with clinical symptoms of localized lung edema and pulmonary hyperemia were observed after the Pm infection. The stained lung showed an inflammation reaction with large inflammatory cells infiltrating and destroying the alveolar structure in the Pm infection groups, whereas the lung tissue in the CK group showed a normal morphology and structure. No Pm was detected in the tissue of the CK group, whereas the Pm infection groups showed a positive DNA band by PCR method (Figure A1).

### 3.2. Small RNA Sequencing and Identification

**Figure 3:**
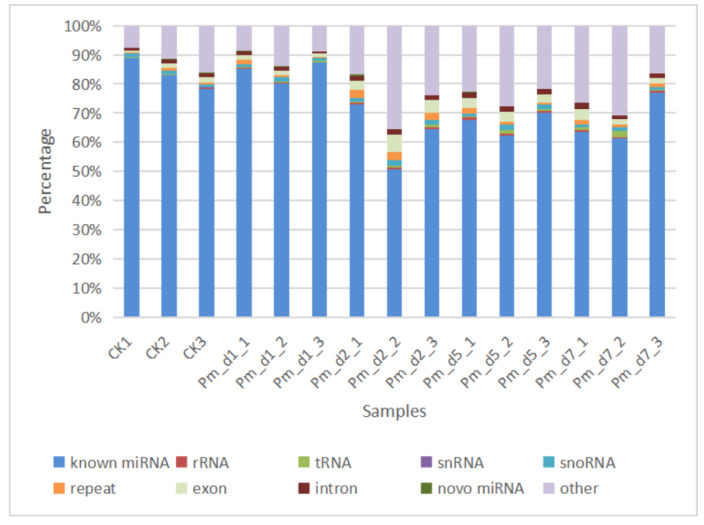
Different categories of small RNAs results are shown for each goat involved in the study (*n* = 3).

**Figure A2:**
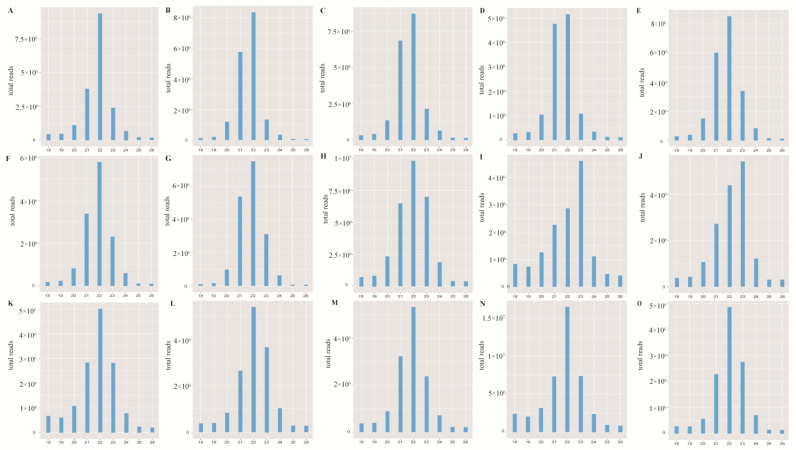
Length distribution of the read counts of sequencing data for small RNAs are shown for each goat involved in the study. A-O indicate the sample of CK1, CK2, CK3, Pm_d1_1, Pm_d1_2, Pm_d1_3, Pm_d2_1, Pm_d2_2, Pm_d2_3, Pm_d5_1, Pm_d5_2, Pm_d5_3, Pm_d7_1, Pm_d7_2 and Pm_d7_3, respectively.

The total raw read count from the sequencing of 15 libraries was 365,668,447 reads, with an average of 24,377,896 reads per sample. After adaptor removal and quality filtering, the resulting high-quality data accounted for 75.87% to 94.13% of the raw counts and was used for further analysis (Table A2). Out of this number, the reads that were successfully mapped to the goat genome were further classified into different small RNA categories (Figure 3). The known miRNAs obtained by mapping to the miRBase, which was used to find statistically differentially expressed miRNAs. The majority of reads for small RNAs were 20–25 nt in length, with a sharp peak at 22 nt (Figure A2).

### 3.3. Characterization of the Lung Transcriptome

In total, 15 libraries were generated from the RNA samples of the Pm-infected goats after 1, 2, 5 and 7 days, and the control groups were sequenced. RNA sequencing generated 2,832,285,308 raw paired reads with an average of 1.89 billion reads per library. Clean reads were obtained for each sample after removing the adaptor and low-quality reads. The alignment of clean reads showed that more than 90.71% (124,374,277) were mapped to the goat genome. Of the mapped reads, an average of 93.03% mapped to unique positions. Only reads that mapped to unique positions were used in further analysis (Table 2).

### 3.4. Effects of Pm on the Expression of mRNAs and miRNAs in the Lung of Goats

**Figure 4:**
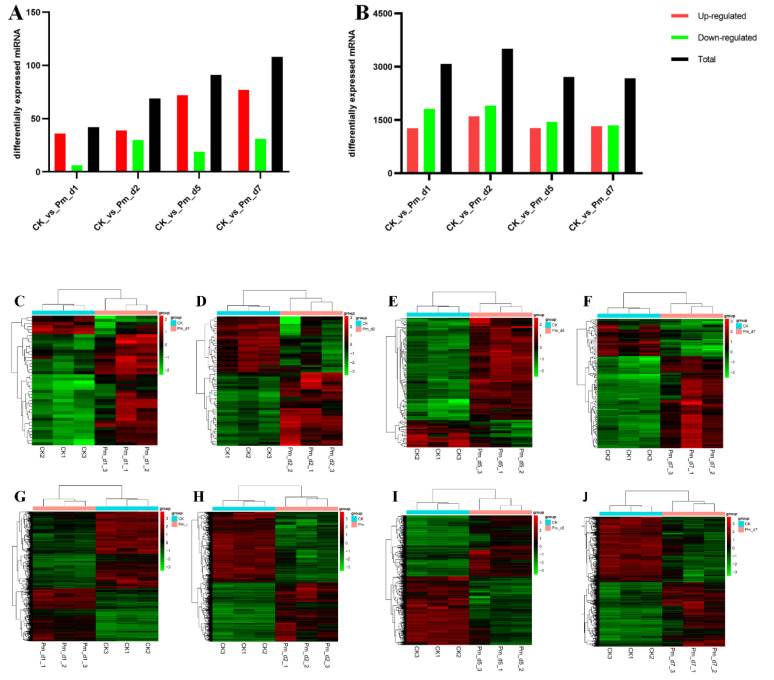
Differentially expressed miRNA and mRNA in the lung tissues of Pm–challenged goats compared with the CK group. (**A**,**B**) Numbers of differentially–expressed miRNA and mRNA molecules. (**C**–**J**) Hierarchical clustering analysis heatmap of the differentially-expressed miRNA and mRNA molecules in the Pm infected group compared with the CK group, respectively. CK_vs_Pm_d1, CK_vs_Pm_d2, CK_vs_Pm_d5 and CK_vs_Pm_d7 indicate the goats infected with Pm compared to CK group 1 day, 2 days, 5 days and 7 days after Pm infection, respectively.

During the Pm infection of the goats, compared with the control group, there were 3080, 3508, 2716 and 2675 differentially expressed genes and 42, 69, 91 and 108 significantly expressed miRNAs (*p* < 0.05) in the Pm_d1, Pm_d2, Pm_d5 and Pm_d7 groups, respectively (Figure 4A,B). The heatmap of the differentially expressed miRNAs and genes is shown in Figure 4C–J.

### 3.5. Functional Annotation of Differentially Expressed Genes

**Figure 5:**
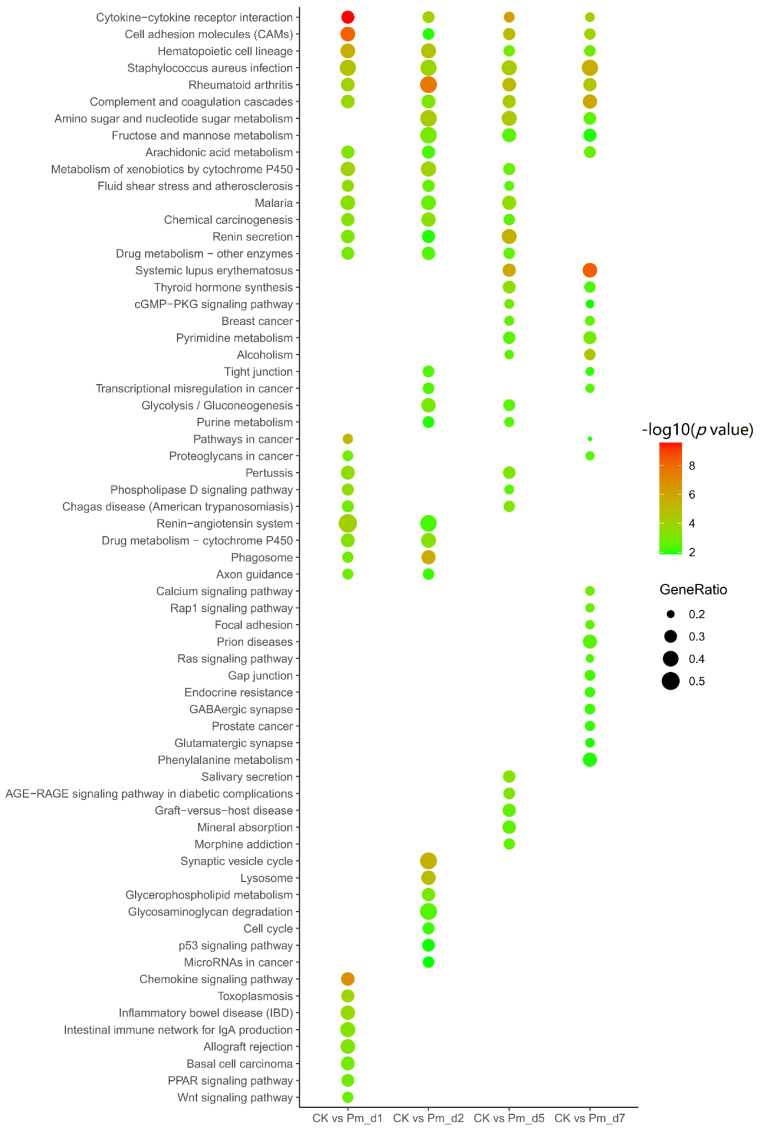
Top 30 enriched KEGG pathways for DEGs in the lung tissues of Pm–challenged goats 1 day, 2 days, 5 days and 7 days compared with the CK group. The x-axis indicates the compared groups, while the names of the GO terms are shown on the y-axis. The size and color of the dot indicate the gene ratio (the number of differentially expressed genes/total genes for each GO term) and the −log_10_ (*p*-value) (Fisher’s exact test), respectively.

**Figure A3:**
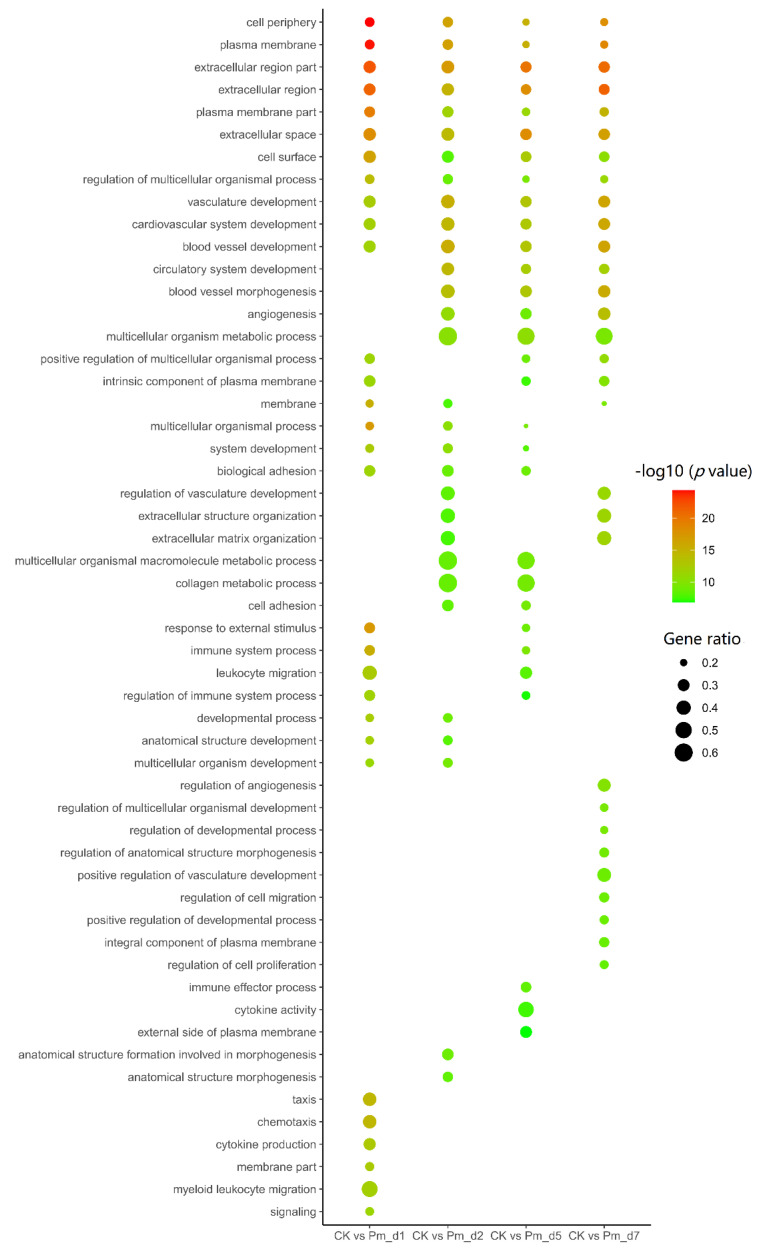
Top 30 enriched GO terms for DEGs in the lung tissues of Pm–challenged goats 1 day, 2 days, 5 days and 7 days compared with the CK group. The x-axis indicates the compared groups, while the names of the GO terms are shown on the y-axis. The size and color of the dot indicate the gene ratio (the number of differentially expressed genes/total genes for each GO term) and the −log_10_ (*p*-value) (Fisher’s exact test), respectively.

GO and KEGG analyses were performed on differentially expressed genes in the different Pm treatment time groups to study the function of differentially expressed genes. The enriched GO terms are listed in Figure A3. The cellular component of the GO-term-enriched analysis showed that cell periphery, plasma membrane, extracellular region, plasma membrane part, extracellular space and cell surface were affected during the Pm infection. Leukocyte migration, myeloid leukocyte migration, leukocyte chemotaxis, regulation of leukocyte migration were also significantly enriched GO terms. The enriched KEGG pathways are presented in Figure 5. Cytokine–cytokine receptor interaction, complement and coagulation cascades, cell adhesion molecules, phagosome and tight junction, were commonly enriched KEGG pathways by DEGs between the Pm group and the CK group 1 day, 2 days, 5 days and 7 days after Pm infection, respectively.

After Pm infection for 1, 2, 5 and 7 days, the differentially expressed genes were mapped to the human and mouse lung cell marker genes. The results showed mappings of some marker genes of immune cells, such as monocyte cell (CD36 and CD14 for human), macrophage cell (IL10 and CD163 for human and CD163, CCL2, CXCL10 and CD14 for mouse) and granulocyte cell (EPO for human and IL6 for mouse). Other marker genes of different cell types in the lung tissue were also mapped. The primary five mapped gene cells were epithelial cell, ciliated cell, lonocyte cell, basal cell and brush cell in the human cell marker; the top five mapped gene cells type in the mouse were ciliated cell, epithelial cell, neuroendocrine cell, basal cell and brush cell (Table A3 and Table A4). Although there were differences between the two species, the trends are similar. Further details are still required through single-cell sequencing.

### 3.6. STEM Analysis of the Common Differentially Expressed miRNAs and Genes

**Figure 6:**
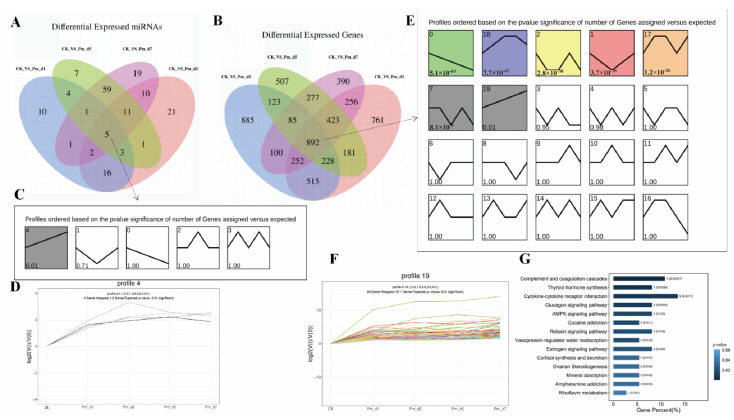
MiRNA and gene analysis by STEM clustering across the five time points. (**A**,**B**) The number of differentially expressed miRNAs and genes identified by each or combination of the different group compared with the CK group. (**C**,**E**) Common differentially expressed miRNAs and genes were assigned to a model profile that most closely matched its temporal expression profile. Statistically significant similar profiles form a cluster of profiles and are shown with the same color. The number in the top and bottom left-hand corner of each profile box is the profile ID and *p*-value. (**D**,**F**) The profile has a statistically significant number of miRNA and genes assigned based on a permutation test. (**G**) The genes in profile 19 significantly enriched the KEGG pathways (*p* < 0.05). CK_vs_Pm_d1, CK_vs_Pm_d2, CK_vs_Pm_d5 and CK_vs_Pm_d7 indicate the goats infected with Pm compared to the CK group 1 day, 2 days, 5 days and 7 days after Pm infection, respectively.

Compared to the CK group, 5 common miRNAs and 892 genes were differentially expressed in the Pm_d1, Pm_d2, Pm_d5 and Pm_d7 groups (Figure 6A,B). These 5 miRNAs and 892 genes were assessed by time-series analysis of the sequencing data using the STEM software to obtain their dynamic expression patterns in the lung tissue of the goats after 1, 2, 5 and 7 days of Pm infection. The STEM clustering tool assigned each miRNA or gene to the model profile closely and matched its temporal expression profile. One and seven statistically significant profiles were generated for miRNAs and genes, respectively (Figure 6C,E). Profile 4 of miRNA and profile 19 of genes are listed in Figure 6D,F, respectively. In addition, 69 genes in profile 19 were used for the KEGG pathway analysis (Table A5 and Table A6). The principal three significantly enriched KEGG pathways included complement and coagulation cascades, thyroid hormone synthesis and cytokine–cytokine receptor interaction (Figure 6G).

### 3.7. Reverse Transcription Quantitative-PCR (RT-qPCR) Verification

**Figure 7:**
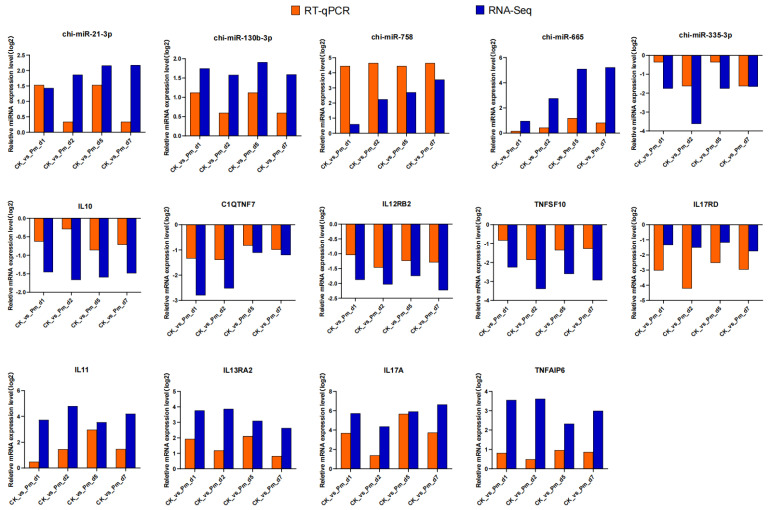
The fold change trend analyses of differentially expressed miRNAs (chi–miR–21–3p, chi–miR–130b–3p, chi–miR–758, chi–miR–665 and chi–miR–335–3p) and genes (IL10, C1QTNF7, IL12RB2, TNFSF10, IL17RD, IL11, IL13RA2, IL17A and TNFAIP6) obtained by qPCR and RNA-seq. CK_vs_Pm_d1, CK_vs_Pm_d2, CK_vs_Pm_d5 and CK_vs_Pm_d7 indicates goats infected with Pm compared with the CK group 1, 2, 5 and 7 days after Pm infection, respectively. The y-axis indicates log_2_FC for RNA-Seq data and log_2_(2^−^^△△ct^) for qPCR data, respectively.

RT-qPCR was used to validate the expression levels of 9 DEGs (IL10, C1QTNF7, IL12RB2, TNFSF10, IL17RD, IL11, IL13RA2, IL17A and TNFAIP6) and 5 DE miRNAs (chi–miR–21–3p, chi–miR–130b–3p, chi–miR–758, chi–miR–665 and chi–miR–335–3p) identified in this study (Figure 7). The expression trends of selected miRNAs and genes generally corresponded with the results from sequencing.

### 3.8. MiRNAs-mRNA Network Identification

**Figure 8:**
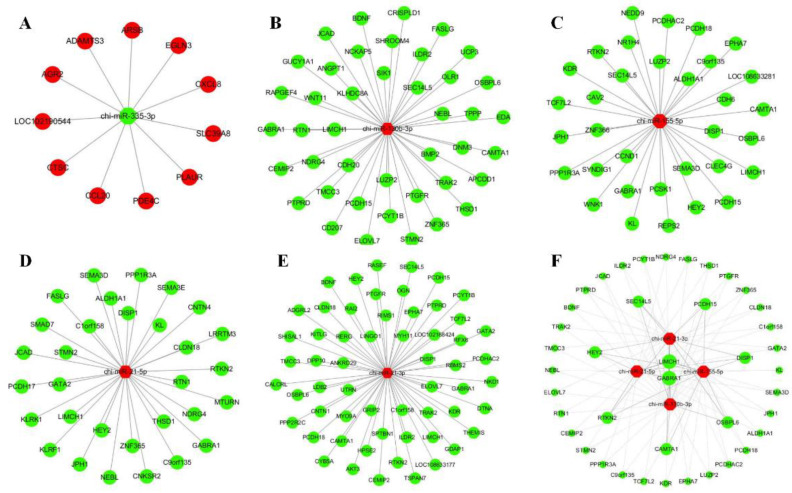
The regulatory networks of target differential expression genes and miRNAs. (**A**–**E**) shows the five common differentially expressed miRNAs–targets gene network (chi–miR–335–3p, chi–miR130b–3p, chi–miR–155–5p, chi–miR–21–5p and chi–miR–21–3p), respectively. (**F**) shows the target genes were commonly targeted by two, three or four up-regulated miRNAs. Red and blue represent up–and down–regulated genes or miRNAs, hexagons and circles indicate the differentially expressed miRNA and mRNA molecules, respectively.

The target relationship between the 5 and 892 common differentially expressed miRNAs and mRNAs was analyzed. Compared with the CK group, 1 and 4 common miRNAs were down– and up–regulated after Pm infection, respectively. The miRNA-target gene networks were constructed for five miRNAs based on the reverse expression change pattern in the corresponding group. Finally, the summary and profile of the results were merged and are shown in Figure 8. Four up-regulated miRNAs commonly targeted the LIMCH1 and GABRA1 genes.

## 4. Discussion

Respiratory disease is a severe ailment in goat breeding in tropical and subtropical areas. The disease, which causes enormous economic losses in the goat breeding industry, is caused by Pm. This study aimed to explore the pathogenic mechanisms of serotype D Pm–infected Hainan black goats. A previous study reported that Pm–challenged goats showed changes at the transcriptome level in the goat lung [10]. Therefore, this study explored the effects of different infection periods on the miRNA and mRNA levels of goats. In addition, the changes of the clinical symptoms, body temperature and lung histopathology were monitored. Three goats died due to acute and fulminating conditions, while Pm triggered an extreme inflammatory response in the other goats. The lung lesions and HE staining results confirmed the inflammatory response (Figure 2). This typical symptom was also observed in another study [5]. The body temperature returned to normal 6 days after the Pm challenge (Table 1). One possible reason is that the lung tissue lesions were more severe and acute in the first 5 days after the Pm infection. Correspondingly, the morphology of the lung tissue lesions gradually decreased in the samples collected 5 to 7 days after Pm infection (Figure 2). Additionally, fewer DEGs were noticed in the 5 to 7 period after Pm infection than after 1 and 2 days (Figure 4B).

The HE stained figures of the lung lesion showed several macrophages, monocytes and a marked neutrophil response at the site of the Pm infection lesion. Marker genes for these three cell types were also among the differentially expressed genes after Pm infection. CD163 is a macrophage-specific protein and the upregulated expression of this receptor is one of the significant changes in the macrophage switch to alternatively activated phenotypes in inflammation [18]. Compared with the CK group, a high CD163 expression was observed after Pm infection after 1, 2, 5 and 7 days in this study. This was a characteristic of the lung tissue responding to pathogen-induced inflammation. The pulmonary macrophages are responsible primarily for recognizing pathogens and protecting the host by initiating inflammation and clearing organisms from the bronchioles and alveoli. It is practical to observe the pulmonary macrophages that phagocytose or kill bacteria, as a part of pathogenesis [19]. A marked neutrophil response was observed in Pm-infected adult cattle [20]. Consistent with this study, a large number of neutrophil infiltration was also observed in the lung lesion of Pm-infected goats in the current study. Therefore, the neutrophils play a role in response to pathogen infection in goats. However, inflammatory cell responses have been reported to be regulated by cytokines [21]. The differentially expressed cytokines such as IL6, IL10, IL11, IL15, TNFSF10, TNFRSF6B and TNFAIP6 were detected in the lung of goats after Pm infection in this study. The expression of some of these was further validated by qPCR (Figure 7).

Enrichment analysis of the DEGs in the different groups indicated that many immune GO terms and KEGG pathways were enriched. The GO enrichment results showed that several cellular components and leukocytes migration terms were enriched. The results indicated that Pm infection affected the cellular component. ‘Leukocytes migration’ GO terms may have facilitated leukocytes migrating to the lesions in the lung to eliminate the inflammatory trigger and contribute to tissue repair [22]. This process is initiated mainly by pathogen-associated molecular patterns released by the invading pathogens and damage-associated molecular patterns derived from damaged and/or dead cells or in response to tissue and/or cellular injury [23]. In addition, cytokine production and activity were also enriched. The cytokines may be released by effector T cells and macrophages to trigger leukocytes’ recruitment. Once in the interstitial tissue, leukocytes can exhibit multiple forms and numerous cellular and molecular regulatory mechanisms [24,25]. Leukocyte migration through activated venular walls is a fundamental immune response. The primary step in leukocyte migration is the establishment of adhesive interactions between leukocytes and endothelial cells of postcapillary venular walls close to inflamed tissues [26]. This critical association is mediated by an array of cell-surface adhesion molecules. In our study, the DEGs significantly enriched cell adhesion molecules (CAMs) as a KEGG pathway after the Pm infection. This may indicated that leukocytes are leaving vessels and entering the inflammary tissue to destroy pathogens. In addition, the activation of endothelial cells is a decisive step in this process and can be induced by histamines, platelet-activating factors and cytokines [27]. The enriched GO term ‘cytokines production’ strongly suggests that cytokines contributed to inducing endothelial cell activation in this study. Dysregulation of inflammatory cytokines is likely a common factor in developing Pm-caused pneumonia.

In the enriched KEGG pathways, multiple immune signaling pathways were significantly enriched. The cytokine–cytokine receptor interaction pathway, as one of the most enriched KEGG pathways (Figure 5), was also influenced by avian pathogenic *Escherichia coli* (APEC) infection in the chicken trachea [28] and was involved in the rehabilitation process after respiratory syncytial virus infection [29]. In addition, the DEGs were enriched in the activated complement and coagulation cascades KEGG pathway, suggesting that Pm infection can activate the complement and coagulation cascades. Rapid activation of the complement and coagulation cascades protects against invading pathogens and limits further bleeding. When these cascades are over-amplified by severe injury, the imbalanced response rapidly leads to the destruction rather than the repair of the injured tissue. This exaggerated and disordered response can result in multi-organ dysfunction syndrome (MODS), which is frequently fatal [30]. These mechanisms are the likely reasons that caused the goat lung lesion and acute death 12 h after Pm infection. As an enriched KEGG pathway, the phagosome destroys and digests pathogens. Tight junctions form and regulate the paracellular barrier between epithelial and endothelial cell sheets. These sheets were also affected by the Pm infection since the lung epithelial barrier damage facilitated the Pm invasion. Praveena et al. reported that Pm infection caused ultrastructural changes in alveolar epithelia of the lungs [31]. Moreover, disruption of the lung epithelial barrier structure by Pm was also reflected by HE staining in our study.

Differentially expressed miRNAs increased gradually from 1, 2, 5 and 7 days after infection (Figure 4A). MiRNAs are mainly transcriptional and translational level regulators and the miRNAs change process is slow. Due to the innate immune response in the primary period, there was greater regulation of the inflammation reaction by other factors, such as various transcription factors and signaling pathways, and they were less dependent on miRNAs regulation. Compared with the CK group, five miRNAs were commonly differentially expressed in the Pm-infected animals after 1, 2, 5 and 7 days. Chi-miR-335-3p was the only significantly down-regulated miRNAs in the Pm-infected group. In the lung adenocarcinoma cells, miR-335-3p was targeted and down-regulated by the Coatomer Protein Complex Subunit beta 2 (COPB2) Gene [32]. MiR-335-5p was down-regulated in the Parenchymal Lung Fibroblasts of smokers because cigarette smoking caused lung inflammation and tissue damage [33]. In addition, miR-335-3p has lower expression in immune diseases such as T-cell acute lymphoblastic leukemia [34] and childhood acute lymphoblastic leukemia [35]. Therefore, miR-335-3p may regulate multiple target genes and other regulatory factors, such as lncRNA [36], which are commonly involved in the host inflammation response reaction.

MiR-155-5p is a multifunctional miRNA enriched in the cells of the immune system and involved in the immune response. Chi-miR-155-5p was up-regulated in the Pm-infected groups in this study. Aziza’s analysis indicated that miR-155 could regulate chemokine production and pro-inflammatory chemokine receptor expression, contributing to Rheumatoid Arthritis pathogenesis and promoting inflammatory cell recruitment and retention in the RA synovium [37]. Therefore, we speculated that the higher expression of chi-miR-155-5p was due to the Pm infection. Pm infection induced the release of cytokines, which promoted many inflammatory cells such as monocytes and macrophages. MiR-155-5p is highly expressed in activated B- and T-cells and monocytes/macrophages [38]. This may be the reason for the up-regulation of miR-155-5p in the lung of the Pm-infected goats.

Chi-miR-21-3p and Chi-miR-21-5p are derived from opposite arms of the same pre-miRNA. Although both were up-regulated in the Pm infected groups, the expression of chi-miR-21-5p was more than 35,000 times that of chi-miR-21-3p. This study’s result agrees with the increased expression of miR-21-5p observed after *Mycobacterium tuberculosis* infection of RAW264.7 and THP-1 cells [39]. MiR-21-5p was also showed elevated expression in a murine macrophage cell line by activation of NF-κB -mediated inflammation with LPS stimulation [40]. Therefore, we speculated that the high expression of chi-miR-21 regulated the Pm survival and inflammatory responses through multiple target genes and involved immune-related signaling pathways in the proliferated immune cells of the lung.

MiR-130b-3p has been reported to be associated with the immune system in the lung by its target genes [41]. MiR-130b-3p was up-regulated after the *Mycoplasma gallisepticum*-infected chicken by activating the PTEN/PI3K/AKT/NF-κB pathway to defend against pathogen invasion [42]. Consistent with this study, miR-130b-3p was also up-regulated after Pm infection, maybe through the same pathway. However, this conjecture needs further validation.

## 5. Conclusions

In conclusion, we characterized the histopathlogy, miRNA and mRNA expression dynamics in lung of goats during infected with Pm for 1,2, 5 and 7 days and the clinical symptoms of animals. The infected lung tissue showed characteristics of suppurative pneumonia with inflammatory cells infiltration and lung structure destruction. Meanwhile, compared with the CK group, there were 3080, 3508, 2716 and 2675 differentially expressed genes, respectively, in the Pm_d1, Pm_d2, Pm_d5 and Pm_d7 groups, and 42, 69, 91 and 108 miRNAs were significantly expressed. The differentially expressed miRNAs and genes commonly involved and regulated the host immune reaction to the Pm infection. Finally, the potential mechanism of the host lesions in the lung caused by Pm was discussed in the context of the hosts’ genes and miRNAs. Although there were DEGs mapped to the marker genes of the immune cells, further details are still required through single-cell sequencing in the future.

## Figures and Tables

**Table 1:** Body temperature of goats recorded for 7 days after Pm infection.

Group	CK	Pm	*p* Value
Before Pm infection	39.73 ± 0.40	39.46 ± 0.20	0.7042
1 day postinfection	39.63 ± 0.49 ^a^	40.68 ± 0.18 ^b^	0.02668
2 days postinfection	39.33 ± 0.32 ^a^	40.40 ± 0.19 ^b^	0.02734
3 days postinfection	39.50 ± 0.19 ^a^	40.61 ± 0.18 ^b^	0.01764
4 days postinfection	39.18 ± 0.29 ^a^	40.37 ± 0.16 ^b^	0.01056
5 days postinfection	38.83 ± 0.13 ^a^	39.40 ± 0.12 ^b^	0.03634
6 days postinfection	39.13 ± 0.10	39.23 ± 0.19	0.8817
7 days postinfection	39.28 ± 0.13	38.90 ± 0.34	0.3807

CK—control group; Pm—intratracheally infected with Pm HN01 group; values are expressed as mean ± SE. ^a,b^ means with different superscripts within the same day column differ significantly (*p* < 0.05).

**Table 2:** Data showing statistical results of RNA-seq for goat lung tissues in the different groups.

Sample	Raw Reads No.	Clean Reads (%)	Mapped Ratio	Uniquely Mapped
CK1	159,020,568	138,839,006 (87.3%)	133,715,619 (96.31%)	128,166,719 (95.85%)
CK2	159,962,714	141,450,102 (88.42%)	135,420,175 (95.74%)	129,758,363 (95.82%)
CK3	140,993,056	122,409,074 (86.81%)	116,539,944 (95.21%)	111,497,935 (95.67%)
Pm_d1_1	141,775,034	125,293,186 (88.37%)	115,772,223 (92.40%)	111,453,233 (96.27%)
Pm_d1_2	128,145,548	112,676,452 (87.92%)	107,539,109 (95.44%)	103,451,962 (96.20%)
Pm_d1_3	161,949,226	135,318,804 (83.55%)	127,410,673 (94.16%)	120,241,761 (94.37%)
Pm_d2_1	395,155,378	334,957,476 (84.76%)	323,192,095 (96.49%)	311,402,497 (96.35%)
Pm_d2_2	141,061,468	125,644,030 (89.07%)	119,625,239 (95.21%)	114,903,208 (96.05%)
Pm_d2_3	169,812,994	139,324,474 (82.04%)	132,020,127 (94.76%)	124,209,044 (94.08%)
Pm_d5_1	161,727,556	138,684,536 (85.75%)	132,145,733 (95.29%)	125,104,988 (94.67%)
Pm_d5_2	178,729,546	137,668,850 (77.02%)	131,890,274 (95.80%)	88,688,766 (67.24%)
Pm_d5_3	158,213,718	110,834,652 (70.05%)	101,373,977 (91.46%)	90,251,977 (89.03%)
Pm_d7_1	427,066,934	344,170,438 (80.58%)	325,241,228 (94.50%)	310,845,471 (95.57%)
Pm_d7_2	152,733,316	137,107,364 (89.76%)	124,374,277 (90.71%)	117,538,318 (94.50%)
Pm_d7_3	155,938,252	134,645,188 (86.34%)	125,868,391 (93.48%)	118,062,150 (93.80%)

## Data Availability

All RNA and small RNA sequencing data generated in this study are available in the NIH short read archive (Accession Number: SUB11040106 and SUB10678249, Bioproject: PRJNA804497 and PRJNA781464). All the analyzed datasets in the current study are available from the corresponding author on reasonable request.

## Abbreviations

The following abbreviations are used in this manuscript:

Pm	Pasteurella multocida
miRNA	microRNA
mRNA	messenger RNA
CD36	CD36 Molecule
CD14	CD14 Molecule
IL10	Interleukin 10
CD163	CD163 Molecule
CCL2	C-C Motif Chemokine Ligand 2
EPO	Erythropoietin
IL6	Interleukin 6
Ca	Calcium
Mg	Magnesium
P	Phosphorus
HTS	High-throughput sequencing
DEGs	differentially expressed genes
IgY	immunoglobulin Y
IgA	immunoglobulin A
NPTr	newborn pig tracheal epithelial
PCR	polymerase chain reaction
CFU	Colony-Forming Units
PBS	phosphate-buffered saline
CK	control group
SE	Standard error
NcRNAs	non-coding RNA
STEM	Short Time-series Expression Miner
KEGG	Kyoto Encyclopedia of Genes and Genomes
GO	Gene Ontology
qPCR	Quantitative Real-time polymerase chain reaction
C1QTNF7	C1q And TNF Related 7
IL12RB2	Interleukin 12 Receptor Subunit Beta 2
TNFSF10	TNF Superfamily Member 10
IL17RD	Interleukin 17 Receptor D
IL11	Interleukin 11
IL13RA2	Interleukin 13 Receptor Subunit Alpha 2
IL17A	Interleukin 17A
TNFAIP6	TNF Alpha Induced Protein 6
CXCL10	C-X-C Motif Chemokine Ligand 10
LIMCH1	LIM And Calponin Homology Domains 1
GABRA1	Gamma-Aminobutyric Acid Type A Receptor Subunit Alpha1
HE	Haemotoxylin-eosin
CAMs	cell adhesion molecules
APEC	avian pathogenic Escherichia coli
MODS	multi-organ dysfunction syndrome
COPB2	Coatomer Protein Complex Subunit beta 2
lncRNA	long non-coding RNA
FC	fold change

## Appendix A

**Table A1:** Primers used for qPCR analyses.

RNAs	Sequence (5′-3′)	Tm (°C)	Product (bp)
mRNAs			
IL10_F	ACAGCCCCGGTACATTC	63.9	193
IL10_R	TTGACCCTCCTCTGCCT	64.6	
C1QTNF7_F	CTGCCTGGAGTGTGTCG	64.9	88
C1QTNF7_R	GCAGCCTTTCTTCTGGGT	64.8	
IL12RB2_F	CTTGGCTGGTTGTGGAGT	64.7	150
IL12RB2_R	TGCTTTGTGCTGGGAGTT	64.7	
TNFSF10_F	ACATGGTGGAAGTAAGGCA	63.7	101
TNFSF10_R	CAGGGAGCTGAGTGGTAAA	63.8	
IL17RD_F	TTCCCCAGCTCATCTCTC	62.9	169
IL17RD_R	GCCTTTCACAGTTCCACAT	63	
IL11_F	CTGCTGAAAACTCGGCTGT	65.6	104
IL11_R	CCTCTGACTGCCAAACTCC	65.1	
IL13RA2_F	CGTGCGTTAGAGTGTATTGATT	63.5	196
IL13RA2_R	TAAGGTAGTCTGGTGGCAAAG	64.3	
IL17A_F	GCAATGAGGACCCTGAGA	63.5	119
IL17A_R	TTGCTGGATGGTGACAGA	63.4	
TNFAIP6_F	TTAAATCTCCAGGCTTCCC	61.5	144
TNFAIP6_R	CAAAATAGTCAGCCAAGCAA	61.5	
GAPDH_F	TCCTGCGCTCTCTGCTC	66.5	101
GAPDH_R	TCCGTTCACTCCGACCTT	65.5	
miRNAs			
miR-21-3p_F	CAGCCACAAAAGAGCACAAT		
miR-21-3p_R	GGCAACAGCAGTCGATG		
miR-21-3p_P	FAM-TTCAGGAGACAACAGG-m		
miR-130b-3p_F	CAGCCACAAAAGAGCACAAT		
miR-130b_R	GACGCAGTGCAATGATGAAA		
miR-130b_P	FAM-TTCAGGAGACAACAGG-m		
miR-758_F	CAGCCACAAAAGAGCACAAT		
miR-758_R	GGTTTGTGACCTGGTCCA		
miR-758_P	FAM-TTCAGGAGACAACAGG-m		
miR-665_R	GGACCAGTAGGCCGAG		
miR-665_F	CAGCCACAAAAGAGCACAAT		
miR-665_P	FAM-TTCAGGAGACAACAGG-m		
miR-335-3p_F	CAGCCACAAAAGAGCACAAT		
miR-335-3p_R	AGGGGCTTTTTCATTATTGCTC		
miR-335-3p_P	FAM-TTCAGGAGACAACAGG-m		
5srRNA_F	CAGCCACAAAAGAGCACAAT		
5srRNA_R	GAGACCGCCTGGGAATAC		
5srRNA_P	FAM-TTCAGGAGACAACAGG-MGB		
*Pasteurella multocida* validation PCR primers			
dcfB_F	TTACAAAAGAAAGACTAGGAGCCC		647
dcfB_R	CATCTACCCACTCAACCATATCAG		

F, R and P represent the forward primers, reverse primers and probe primers, respectively.

**Table A2:** Characterization of sequencing data for small RNAs from the lungs of the goats in each group.

Sample	Raw Reads No.	Clean Reads (%)
CK1	19,573,455	18,426,013 (94.14%)
CK2	24,109,866	22,147,178 (91.86%)
CK3	16,111,570	14,330,716 (88.95%)
Pm_d1_1	23,944,345	22,109,701 (92.34%)
Pm_d1_2	16,138,271	14,941,398 (92.58%)
Pm_d1_3	21,509,388	19,501,005 (90.66%)
Pm_d2_1	36,063,670	32,573,615 (90.32%)
Pm_d2_2	21,280,232	17,458,245 (82.04%)
Pm_d2_3	25,959,057	19,695,559 (75.87%)
Pm_d5_1	18,812,698	15,403,394 (81.88%)
Pm_d5_2	24,192,959	18,697,496 (77.28%)
Pm_d5_3	18,188,771	15,673,217 (86,17%)
Pm_d7_1	59,280,166	46,120,696 (77.80%)
Pm_d7_2	18,018,249	16,403,206 (91.04%)
Pm_d7_3	22,485,750	20,188,889 (89.79%)

**Table A3:** The results of DEGs that mapped to the human lung cell marker gene.

Human Lung Cell Marker (Number of the Marker Genes)	CK_vs_Pm_d1	CK_vs_Pm_d2	CK_vs_Pm_d5	CK_vs_Pm_d7
1 epithelial cell (1302)	144	175	141	137
2 ciliated cell (311)	31	43	48	23
3 lonocyte cell (308)	51	70	47	46
4 basal cell (102)	26	27	18	20
5 brush cell (84)	16	15	12	14
6 neuroendocrine cell (84)	16	15	12	14
7 secretory cell (78)	18	18	12	18
8 cancer stem cell (78)	3	3	4	2
9 dendritic cell (27)	1	3	2	4
10 macrophage cell (23)	2	2	3	2
11 endothelial cell (12)	1	1	2	2
12 fibroblast (9)	0	1	0	0
13 T cell (8)	0	0	0	2
14 granulocyte cell (7)	0	1	0	1
15 alveolar cell (4)	1	1	1	1
16 myofibroblast cell (4)	0	0	0	0
17 cancer cell (3)	0	0	0	0
18 monocyte cell (3)	0	1	0	1
19 clara cell (2)	0	0	0	0
20 progenitor cell (2)	0	0	0	0
21 endodem cell (1)	0	0	0	0
22 mesothelial cell (1)	0	0	0	0
23 narural killer cell (1)	0	0	0	0
Total number	310	376	302	287

**Table A4:** The results of DEGs that mapped to the mouse lung cell marker gene.

Mouse Lung Cell Marker (Number of the Marker Genes)	CK_vs_Pm_d1	CK_vs_Pm_d2	CK_vs_Pm_d5	CK_vs_Pm_d7
1 ciliated cell (732)	79	132	122	80
2 epithelial cell (474)	110	113	90	104
3 neuroendocrine cell (470)	78	83	57	55
4 basal cell (356)	39	107	74	95
5 brush cell (tuft cell) (312)	66	68	45	45
6 fibroblast cell (283)	60	84	63	71
7 endothelial cell (152)	52	67	54	57
8 lonocyte cell (110)	21	23	21	20
9 mesenchymal cell (97)	33	39	26	35
10 secretory cell (78)	14	17	14	18
11 alveolar cell (64)	22	23	22	19
12 immune cell (64)	22	22	12	7
13 myeloid cell (64)	22	22	12	7
14 clara cell (51)	13	12	12	10
15 myofibroblast cell (44)	15	16	11	11
16 pericyte cell (39)	14	10	11	8
17 smooth muscle cell (36)	13	14	10	9
18 macrophage cell (21)	2	2	2	2
19 lipofibroblast cell (15)	3	3	0	1
20 granulocyte cell (14)	1	1	1	0
21 progenitor cell (14)	2	7	4	5
22 mesothelial cell (10)	3	3	3	4
23 cancer stem cell (8)	1	1	1	1
24 T cell (8)	1	1	0	1
25 dendritic cell (4)	0	0	0	0
26 endoderm cell (1)	0	0	0	0
27 goblet cell (1)	0	0	0	0
Total number	686	870	667	665

**Table A5:** The differentially expressed genes listed in profile 19.

Ensembl ID	Gene Name	Ensembl ID	Gene Name
ENSCHIG00000000151	CRELD2	ENSCHIG00000019736	LBP
ENSCHIG00000000508	ENPP3	ENSCHIG00000020376	MDFI
ENSCHIG00000000977	SLC2A10	ENSCHIG00000020647	SLC5A5
ENSCHIG00000004432	TTC39A	ENSCHIG00000020739	STEAP2
ENSCHIG00000004710	GJB2	ENSCHIG00000021027	ACAN
ENSCHIG00000005302	P2RY6	ENSCHIG00000021212	CPXM1
ENSCHIG00000005671	STEAP1	ENSCHIG00000022340	F11
ENSCHIG00000006570	UGGT2	ENSCHIG00000022648	LOC102180889
ENSCHIG00000006701	FRK	ENSCHIG00000023001	UNC5A
ENSCHIG00000006757	SERPINB7	ENSCHIG00000023479	C1R
ENSCHIG00000007788	CHST4	ENSCHIG00000023717	SLC7A10
ENSCHIG00000007961	EID2	ENSCHIG00000023885	SLC39A14
ENSCHIG00000008233	FAT2	ENSCHIG00000023922	SLC25A15
ENSCHIG00000008358	IL17F	ENSCHIG00000024682	PRRX2
ENSCHIG00000008595	EPOR	ENSCHIG00000024749	MMP23B
ENSCHIG00000008665	PLPPR2	ENSCHIG00000025275	CPT1C
ENSCHIG00000008753	CREB3L1	ENSCHIG00000025398	IL19
ENSCHIG00000008980	LOC102171051	ENSCHIG00000025478	CREB3L4
ENSCHIG00000009050	PLA2G4F	ENSCHIG00000025500	TMEM106C
ENSCHIG00000009912	MYBPC2	ENSCHIG00000025636	ASPG
ENSCHIG00000010028	LOC102169690	ENSCHIG00000026053	RND1
ENSCHIG00000010077	AGP	ENSCHIG00000026468	MMP7
ENSCHIG00000010286	CFHR5	ENSCHIG00000026759	BPIFB2
ENSCHIG00000010991	LOC102174291	ENSCHIG00000026809	PYCR1
ENSCHIG00000011552	ADAM12	ENSCHIG00000027055	ADAMTS2
ENSCHIG00000012064	CHI3L2	ENSCHIG00000027096	LOC102171764
ENSCHIG00000012382	CYP1B1	ENSCHIG00000027222	QPCTL
ENSCHIG00000012922	DUSP5	ENSCHIG00000017490	FAM3B
ENSCHIG00000013053	GALNT12	ENSCHIG00000017697	GMDS
ENSCHIG00000014492	SLC7A4	ENSCHIG00000017941	IMPDH1
ENSCHIG00000015160	TGM5	ENSCHIG00000018332	SYNGR1
ENSCHIG00000016293	MMP2	ENSCHIG00000018403	CCL19
ENSCHIG00000016775	B4GALNT3	ENSCHIG00000018670	NID2
ENSCHIG00000017087	SPDEF	ENSCHIG00000019003	TNFSF13
ENSCHIG00000017285	SH3PXD2B		

**Table A6:** KEGG pathways analysis results of DEGs in profile 19.

Pathway ID	Pathway Name	DEG Number	Class	*p* Value	FDR	Total Number
ko04610	Complement and coagulation cascades	4	Organismal Systems	0.000366826	0.042185	82
ko04918	Thyroid hormone synthesis	3	Organismal Systems	0.003758384	0.2161071	75
ko04060	Cytokine-cytokine receptor interaction	5	Environmental Information Processing	0.007733519	0.2571427	298
ko04922	Glucagon signaling pathway	3	Organismal Systems	0.009324506	0.2571427	104
ko04152	AMPK signaling pathway	3	Environmental Information Processing	0.01499447	0.2571427	124
ko05030	Cocaine addiction	2	Human Diseases	0.01652974	0.2571427	47
ko04926	Relaxin signaling pathway	3	Organismal Systems	0.01770463	0.2571427	132
ko04962	Vasopressin-regulated water reabsorption	2	Organismal Systems	0.01788819	0.2571427	49
ko04915	Estrogen signaling pathway	3	Organismal Systems	0.02067209	0.2641434	140
ko04927	Cortisol synthesis and secretion	2	Organismal Systems	0.03120647	0.2916459	66
ko04913	Ovarian Steroidogenesis	2	Organismal Systems	0.03296867	0.2916459	68
ko04978	Mineral absorption	2	Organismal Systems	0.03296867	0.2916459	68
ko05031	Amphetamine addiction	2	Human Diseases	0.03296867	0.2916459	68
ko00740	Riboflavin metabolism	1	Metabolism	0.04130254	0.3392709	10
